# Supplementary material for: Identification of a Novel Renal Metastasis Associated CpG-Based DNA Methylation Signature (RMAMS)
Source: Int J Mol Sci. 2022 Sep 23;23(19):11190. doi: 10.3390/ijms231911190 (PMC9569431; doi:10.3390/ijms231911190)
Supplement: Supplementary file 1 [file ijms-23-11190-s001.zip › Figure S1.pdf]

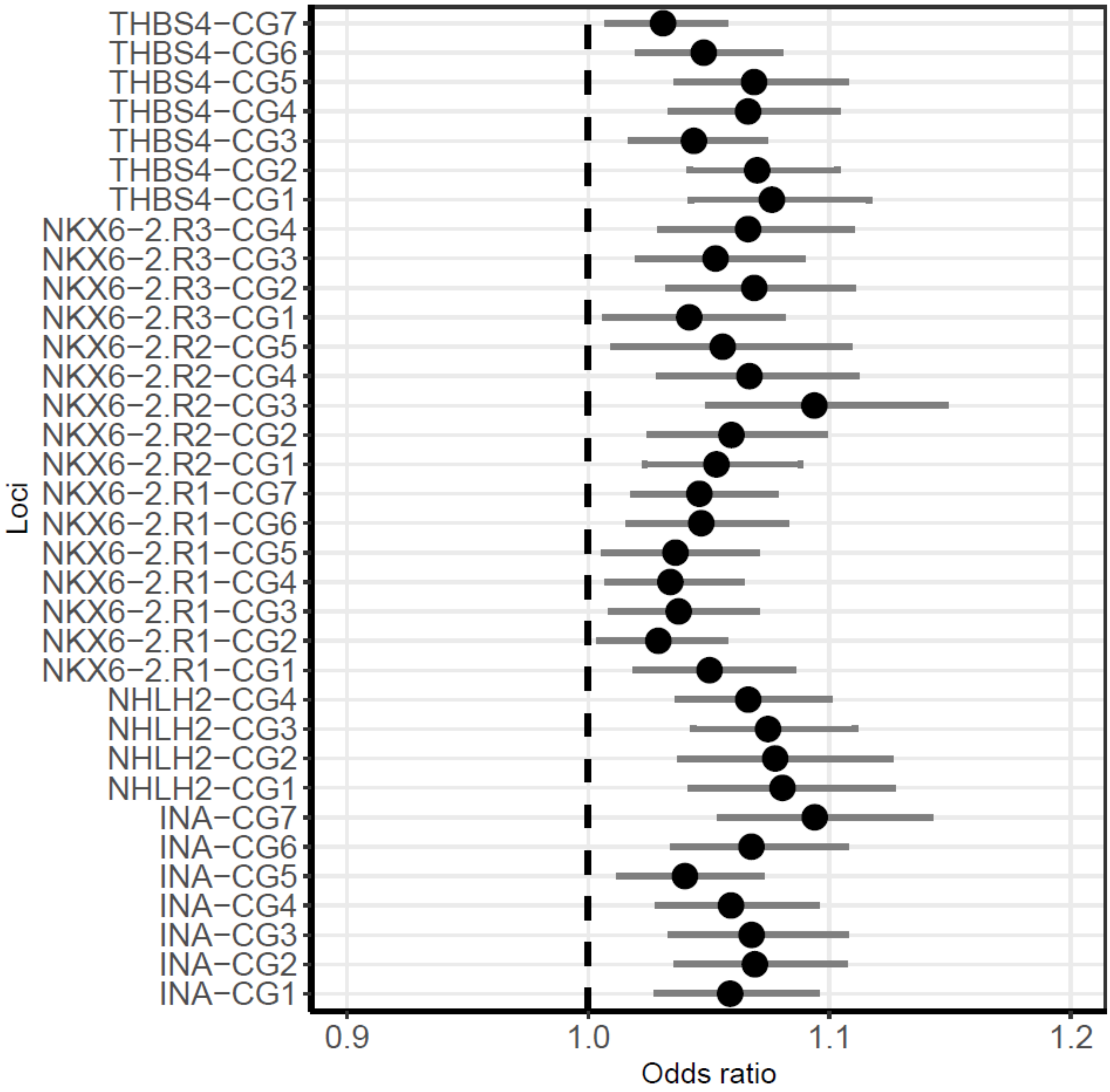

Scheme 1. Forest plot illustration of the statistical comparison of localized primary RCC and renal metastatic tissue methylation.

CpG site-specific data for *NKX6*-2 and previously identified metastatic tissue-associated methylation of the INA, NHLH2, and THBS4 genes [20] is summarized showing the odds ratios (ORs; solid circles) and 95% confidence intervals (CIs; whiskers) obtained by age-adjusted logistic regression analysis. Annotation of the CpG sites refers to Table 1, specifying genomic positions and statistical results.
