# Supplementary material for: Identification of a Novel Renal Metastasis Associated CpG-Based DNA Methylation Signature (RMAMS)
Source: Int J Mol Sci. 2022 Sep 23;23(19):11190. doi: 10.3390/ijms231911190 (PMC9569431; doi:10.3390/ijms231911190)
Supplement: Supplementary file 1 [file ijms-23-11190-s001.zip › Table S1.pdf]

Scheme 1. Patient's clinical and histopathologic characteristics for pyroassays R3-R1.

		Number of patients, n (%)
**Total**		189 (100.0)
**Histologic subtype**	ccRCC	151 (79.9)
	pap. RCC	25 (13.2)
	chrom. RCC	3 (1.6)
	Mixed histology	6 (3.2)
	Other	4 (2.1)
**Gender**	Female	70 (37)
	Male	119 (63)
**Age (years)**	Median	65
	Min-Max	35-91
**Metastasis**	M0	156 (82.5)
	M1	30 (15.9)
	NA	3 (1.6)
**Lymph node status**	N0	168 (88.9)
	N1	16 (8.5)
	NA	5 (2.6)
**Tumor stage**	pT1	11 (5.8)
	pT1a	62 (32.8)
	pT1b	46 (24.3)
	pT2	9 (4.8)
	pT3	5 (2.6)
	pT3a	17 (9.0)
	pT3b	32 (16.9)
	pT3c	4 (2.1)
	pT4	1 (0.5)
	NA	2 (1.1)
**Differentiation**	G1	36 (19.0)
	G1-2	20 (10.6)
	G2	104 (55.0)
	G2-3	10 (5.3)
	G3	18 (9.5)
	NA	1 (0.5)
**State of disease***	Localized	114 (60.3)
	Advanced	68 (36.0)
	NA	7 (3.7)

Abbreviations: ccRCC clear cell renal cell carcinoma (RCC); pap. RCC papillary RCC; chrom. RCC chromophobe RCC; FU follow-up; NA not available

*Localized disease defined as pT ≤ 2, N0, M0; Advanced disease defined as pT ≥ 3 and/or N+, M+
