# Supplementary material for: Identification of a Novel Renal Metastasis Associated CpG-Based DNA Methylation Signature (RMAMS)
Source: Int J Mol Sci. 2022 Sep 23;23(19):11190. doi: 10.3390/ijms231911190 (PMC9569431; doi:10.3390/ijms231911190)
Supplement: Supplementary file 1 [file ijms-23-11190-s001.zip › Table S2.pdf]

Table S2. Primer sequences for pyrosequencing.

**Pyroassay**	**Primer**			**Annealing temperature (°C)**
	Sequencing	Forward	Reverse	
R3	CCTCCCTTACAACCC	GTATTTGGTTTGTTAGAAGGTTTTTTTTTAG	TCCCTTCCCCTCCCTTACAA	57,5
R2	ATTTAGTTTTAGGGGAGATAAAT	TGTTTAATTATATTTTTATTTAAGTTGGATTTATT	CACTCCATAAAAAACCCATT	55
R1	AAATTTAGGGGTAGTGG	TGGATGGAGGTTTGGAAAAGT	AAACTACACCTCAATAAAACC	57,5
