# Supplementary material for: Identification of a Novel Renal Metastasis Associated CpG-Based DNA Methylation Signature (RMAMS)
Source: Int J Mol Sci. 2022 Sep 23;23(19):11190. doi: 10.3390/ijms231911190 (PMC9569431; doi:10.3390/ijms231911190)
Supplement: Supplementary file 1 [file ijms-23-11190-s001.zip › Table S3.pdf]

Table S3. Results of bivariate logistic regression for age adjusted group comparisons of localized and metastasized primary RCC using CpG-site-specific methylation data.

**Pyroassay**	**CpG site**	**Metastatic primary tumor methylation**			
		Mean methylation (%)		OR (95% CI)	p-value
		M0	M+		
R3	CG1	14.9	23.2	1.06 (1.02 – 1.12)	**0.034**
	CG2	15.7	22.6	1.05 (1.01 – 1.09)	0.050
	CG3	14.8	24.4	1.06 (1.02 – 1.11)	**0.034**
	CG4	13.9	24.6	1.07 (1.03 – 1.12)	**0.034**
R2	CG1	14.9	23.5	1.04 (1.00 – 1.07)	0.050
	CG2	12.4	23.1	1.06 (1.02 – 1.10)	**0.034**
	CG3	9.9	18.1	1.06 (1.01 – 1.11)	**0.049**
	CG4	11.9	19.3	1.05 (1.01 – 1.11)	**0.049**
	CG5	6.2	12.4	1.05 (1.01 – 1.12)	0.063
R1	CG1	12.8	22.3	1.05 (1.01 - 1.09)	**0.034**
	CG2	16.1	26.4	1.06 (1.02 – 1.11)	**0.034**
	CG3	16.1	24.7	1.05 (1.01 – 1.10)	**0.034**
	CG4	22.8	27.4	1.02 (0.99 – 1.06)	0.170
	CG5	17.1	20.8	1.02 (0.99 – 1.06)	0.188
	CG6	12.9	20.0	1.04 (1.00 – 1.09)	0.060
	CG7	19.0	24.8	1.04 (1.00 – 1.08)	0.063

OR Odds ratio 95% CI 95% confidence interval CG CpG site
